# Editorial: Clinical Relevance of the Immune-to-Brain and Brain-to-Immune Communications

**DOI:** 10.3389/fnbeh.2018.00336

**Published:** 2019-01-11

**Authors:** Julie Lasselin, Manfred Schedlowski, Mats Lekander, Martin Hadamitzky

**Affiliations:** ^1^Institute of Medical Psychology and Behavioral Immunobiology, University Hospital Essen, Essen, Germany; ^2^Stress Research Institute, Stockholm University, Stockholm, Sweden; ^3^Division of Psychology, Department of Clinical Neuroscience, Karolinska Institutet, Solna, Sweden

**Keywords:** immune system, brain, brain-immune interactions, immunopsychiatry, stress, psychoneuroimmunology, gut-brain axis

Experimental and clinical evidence demonstrates an intense crosstalk among the nervous, endocrine and immune systems (Dantzer, 2018). The central nervous system (CNS) not only has the capacity to affect peripheral immune functions, but is also able to sense and process signals from the peripheral immune system. The bi-directional interaction between the CNS and the peripheral immune system has gained great interest as it can help better understanding disease pathophysiology to improve health and treatment outcomes in patients, and to understand how modifiable life-style factors can be associated with health. In this special issue of *Frontiers in Behavioral Neuroscience, Frontiers in Immunology*, and *Frontiers in Neurology*, we have collected original works and perspectives that provide new insights on the clinical relevance of immune-to-brain and brain-to-immune communications.

## Clinical Relevance of Immune-to-Brain Communications

In order to provide improved understanding of possible underlying mechanisms of **inflammation-associated depression** (Lotrich, 2015), Kotulla et al. studied specific symptoms of negative self-schema (general self-esteem, intolerance of uncertainty, and hopelessness) in response to an inflammatory challenge. Although inflammation lowered mood, negative self-schema was not significantly impacted. This indicates that negative self-schemata might not be a mechanism by which inflammation leads to depression. In their review, Lacourt et al. provide new insights on the mechanisms underlying **chronic fatigue**. They propose that the chronic activation of inflammatory processes at a low-grade state, such as observed in chronic fatigue syndrome (Fletcher et al., 2009) and cancer survivors (Bower, 2007), might contribute to persistent fatigue via two main mechanisms: changes in metabolism, leading to reduced glucose availability and thus to reduced cellular energy; and increased behavioral energy expenditure. Hence, an imbalance between energy expenditure and available resource could lead to persistent fatigue. Koenig et al. investigated the increased **brain vulnerability to psychosocial stress during aging**, which is a risk factor for the development of mental illness (De Kloet et al., 2005). Using young and old rats subjected to psychological novel environment stress and to lipopolysaccharide administration, the authors showed dysregulated pituitary cytokine interactions and brain cell activation in the hypothalamus-pituitary-adrenal axis in old rats. This mechanism could underlie the prolonged and maladaptive response to stressors that is observed in aging.

As highlighted by Tong et al. and Oberstein et al., peripheral immune processes might also be involved in CNS diseases. These authors assessed the peripheral immune profile of two respective patient populations. Tong et al. found that patients with **Neuromyelitis Optica Spectrum Disorders** (NMOSD), a demyelinating disease affecting optic nerve and spinal cord, exhibited higher plasma levels of pro-inflammatory cytokines and of eosinophil chemoattractants, which was, in turn, associated with the number of relapses. The authors suggest that inflammatory activity could contribute to the pathophysiology of NMOSD by increasing eosinophil chemoattractants, and thus favoring eosinophil infiltration, which is a specific feature of NMOSD (Zhang and Verkman, 2013). Oberstein et al. report an increased proportion of Th17 cells in the blood, as well as a significant correlation between blood proportion of regulatory T cells and the levels of Tau in the cerebrospinal fluid, in patients with mild cognitive impairment due to **Alzheimer's Disease** (AD), but not in patients with mild cognitive impairment with negative CSF AD's biomarkers and in healthy controls. This indicates a potential role of the adaptive immune system in the pathophysiology of AD.

The studies from Herz et al. and Tadros et al. reveal the importance of the immune system for the central nervous system during the neonatal period. Based on previous research (e.g., Nazmi et al., 2018), Herz et al. hypothesized that the depletion of peripheral T cells would protect against consequences of **hypoxic-ischemic injury in the neonatal brain** of mice. However, contrary to the hypothesis, the depletion of peripheral T cells led to an exacerbation of the effects of hypoxic-ischemic brain injury in mice neonates, with an increased loss of gray and white matter, along with an increased brain infiltration of innate immune cells. Thus, the observed infiltration of T cells after hypoxic-ischemic injury could actually be protective in neonates. Tadros et al. assessed whether changes in the properties of superficial dorsal horn (SDH) neurons is a mechanism by which an immune challenge during the neonatal period leads to heighten **inflammatory pain** later in life. The authors found that frequency of spontaneous excitatory synaptic currents of SDH neurons was increased in the group of rats having received the neonatal immune challenge compared to saline. This reflects a hyper-excitability of the SDH neuronal network, which could contribute to the altered nociceptive signaling observed after a neonatal immune challenge.

Gut immunity and the gut-brain axis play an important role in modulating CNS functions (Grenham et al., 2011). In their review, Bajic et al. describe how the gut-brain axis is likely to be involved in the **psychological and neurological complications of chemotherapy**. Chemotherapy induces mucositis, associated with an inflammatory response, changes in the gut microbiome, increased intestinal permeability, and damages to the nerves of the myenteric plexus. These changes cause a disruption in the gut-brain axis, which is known to alter mood and cognition. Langgartner et al. show that rectal transplantation of feces from non-stressed mice partially reversed the physiological (thymus atrophy, low-grade inflammation, altered bone homeostasis) and **behavioral (anxiety) effects of chronic psychosocial stress** (induced by housing with a dominant CD-1 mouse). This indicates a potential role of gut microbiota in some of the physiological and behavioral changes induced by chronic psychosocial stress.

## Clinical Relevance of Brain-to-Immune Communications

Dysregulation of peripheral innate immune responses is observed in stress-associated psychiatric disorders, such as major depression (Dowlati et al., 2010). Ambrée et al. studied whether **vulnerability to stress was associated with specific differences in the innate immune system** compared to stress resilience. In mice chronically exposed to social defeat, the authors showed that the innate peripheral and brain immune cells of vulnerable mice (i.e., those that show altered social behavior after social defeat) presented a specific inflammatory profile that was not observed in resilient mice. These findings show a strong interaction between stress vulnerability and immunological consequences.

The **effect of psychosocial stress on immune functions** is studied to a large extent in rodents. In their review, Gimsa et al. propose instead to use **pigs as an animal model** in this field. Many anatomical and physiological characteristics, such as brain anatomy (Lind et al., 2007), stress hormone (cortisol), and immune functions (Meurens et al., 2012), are more similar to humans in pig models than rodents. To support their approach, the authors present data on short-term and long-term effects of social stress in preclinical pig models regarding immunity and neuroendocrine regulation, as well as consequences for health and well-being.

Macrophages are central immune cells contributing to innate and adaptive immunity. In a comprehensive review, Jurberg et al. thoroughly describe how **neurotransmitters and neuroendocrine hormones influence macrophage physiology and functioning**, such as inflammatory responses, macrophage maturation and polarization, and migration. The authors also discuss how these mechanisms could be targeted in the development of therapies for inflammatory diseases and cancer.

Lastly, Willekens et al. build upon the known brain-to-immune communication networks to propose mechanisms by which **mindfulness-based interventions might improve multiple sclerosis (MS) outcomes**. Mindfulness has been shown to improve quality of life, depression and fatigue in MS patients, although the underlying mechanisms remain unknown. The authors provide data supporting immunomodulatory effects of this treatment, and call for the measurement of immunological and neuroimaging biomarkers in studies assessing the effect of mindfulness in MS.

## Conclusion

The articles combined in this special issue highlight the central role of immune-to-brain and brain-to-immune communications in various clinical pathologies, including, but not limited to, depression, anxiety, fatigue, pain, stress-related disorders, neurological diseases, and inflammatory diseases. This special issue also reveals the importance of clinical *and* animal studies, along with review articles reflecting on the current knowledge, in the quest for a better comprehension of the brain-immune mechanisms and their clinical relevance.

## Author Contributions

JL and MH have written the draft of this editorial. MS and ML have provided critical feedback. All authors approve the final version.

### Conflict of Interest Statement

The authors declare that the research was conducted in the absence of any commercial or financial relationships that could be construed as a potential conflict of interest.
